# Current challenges and possible solutions to improve access to care and treatment for hepatitis C infection in Vietnam: a systematic review

**DOI:** 10.1186/s12879-017-2360-6

**Published:** 2017-04-11

**Authors:** Alessandra Berto, Jeremy Day, Nguyen Van Vinh Chau, Guy E. Thwaites, Ngoc Nghiem My, Stephen Baker, Thomas C. Darton

**Affiliations:** 1 Oxford University Clinical Research Unit, Vietnam Wellcome Trust Major Overseas Programme, 764 Vo Van Kiet, District 5, Ho Chi Minh City, Vietnam; 2 Centre for Tropical Medicine and Global Health, Nuffield Department of Clinical Medicine, University of Oxford, Oxford, UK; 3 Hospital for Tropical Diseases, Ho Chi Minh City, Vietnam; 4 The London School of Hygiene and Tropical Medicine, London, UK; 5 Department of Infection, Immunity and Cardiovascular Disease, University of Sheffield, Sheffield, UK

**Keywords:** Hepatitis C virus, Treatment access, Low and middle-income countries, Epidemiology, Vietnam

## Abstract

**Background:**

Hepatitis C infection is a major public health concern in low- and middle-income countries where an estimated 71.1 million individuals are living with chronic infection. The World Health Organization (WHO) has recently released new guidance for hepatitis C virus (HCV) treatment programs, which include improving the access to new direct-acting antiviral agents. In Vietnam, a highly populated middle-income country, the seroprevalence of HCV infection is approximately 4% and multiple genotypes co-circulate in the general population. Here we review what is currently known regarding the epidemiology of HCV in Vietnam and outline options for reducing the significant burden of morbidity and mortality in our setting.

**Methods:**

We performed a systematic review of the currently available literature to evaluate what has been achieved to date with efforts to control HCV infection in Vietnam.

**Results:**

This search retrieved few publications specific to Vietnam indicating a significant gap in baseline epidemiological and public health data. Key knowledge gaps identified included an understanding of the prevalence in specific high-risk groups, characterization of circulating HCV genotypes in the population and likely response to treatment, and the extent to which HCV treatment is available, accessed and utilized.

**Conclusions:**

We conclude that there is an urgent need to perform up to date assessments of HCV disease burden in Vietnam, especially in high-risk groups, in whom incidence is high and cross infection with multiple genotypes is likely to be frequent. Coordinating renewed surveillance measures with forthcoming HCV treatment studies should initiate the traction required to achieve the WHO goal of eliminating HCV as a public health threat by 2030, at least in this region.

## Background

Approximately 71.1 million people worldwide have chronic hepatitis C virus infection, resulting in over 500,000 deaths each year and creating a substantial burden on healthcare services [1–4]. The prevalence of hepatitis C infection is estimated to be 1%, although the highest burden (85%) of chronic infection is in low and middle income countries (LMICs) [1–4].

Hepatitis C virus (HCV), a single-stranded positive RNA virus, can cause individual infection with multiple subtypes, and therefore knowledge of viral genotype is particularly important for predicting virological and clinical treatment response. Genotype distribution varies geographically and is known to be particularly diverse in Southeast Asia [1].

Recently, the treatment options available for chronic hepatitis C infection have undergone a revolution with development of the direct-acting antivirals (DAAs) [5]. Treatment regimes containing drugs from this new class have demonstrated significantly higher rates of sustained virological response (SVR) compared to standard interferon- (IFN) and ribavirin-focused regimes [6, 7]. Additional benefits of this class include shorter treatment durations, oral administration and fewer side-effects. It has been estimated that, with unrestricted access to these costly DAA-containing regimes, more than 90% of HCV infected patients could attain SVR and thus achieve a definitive cure [8].

In May 2016, the World Health Organisation (WHO) launched a new global strategy to eliminate viral hepatitis as public health threat by 2030 [9]. A key priority in these recommendations is to ensure open access to the DAAs [10]. However, individuals living in LMICs, are unlikely to be able to afford regimes containing these new costly treatments [10]. Ongoing efforts to ensure equity of access include the development of generic drug formulations [10, 11].

The Socialist Republic of Vietnam has a population exceeding 93 million people. Since the mid-1980s there have been dramatic improvements in the key development indicators of this Southeast Asian country, including increases in life expectancy, gross-domestic product and expanding state and private healthcare provision [12]. This includes a new national strategy to improve access to viral hepatitis diagnostic and treatment services [12]. To achieve the WHO stated goal of HCV elimination as a public health problem, understanding how the control of HCV in countries such as Vietnam could be improved, is vital. This article aims to review the currently available published literature regarding the epidemiology, virological features and the current treatment options available for hepatitis C infection in Vietnam. We use these data to propose measures which could help in reducing the burden of HCV infections in Vietnam.

## Methods

### Search strategy and selection criteria

**Fig. 1:**
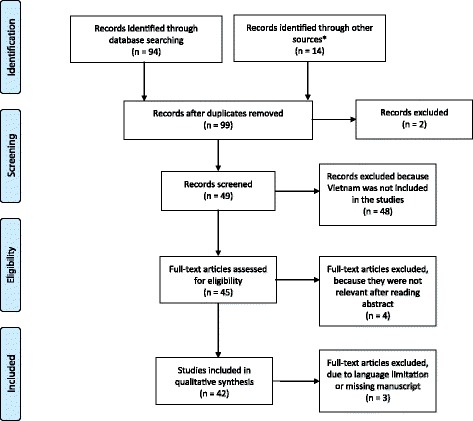
Flow chart of studies included in data synthesis. * Other sources from which records were obtained include *Clinicaltrials.gov* (*n* = 9, see Table 2) and Standard Operating Procedures for the identification, diagnosis and management of Hepatitis C infection published in Vietnamese and identified through web searches (*n* = 5)

We searched PubMed-, MEDLINE- and Embase®-indexed articles for English-Vietnamese languages articles in June 2016, according to the PRISMA guidelines (Fig. 1) [13].

Databases were searched using a combination of the following MeSH terms and keywords, including (Hepatitis C virus OR HCV) AND Vietnam AND (Prevalence OR Epidemiology OR Surve* OR Seroprevalence) (OR hepatitis C). No date restriction was used although included articles/data were restricted to original research articles relating to hepatitis C infection and HCV in Vietnam only, and on those published in either English or Vietnamese. We further searched the clinical trials registry *http:/ClinicalTrial.gov* for treatment trials currently underway or completed in Vietnam using the search term “Hepatitis C” AND “Vietnam”.

After performing the literature searches, one author (AB) extracted and screened the titles and abstracts before selecting and reviewing the full-text articles. The following data were extracted from each article: first author’s name, publication year, country of origin, date of study, type of patient group, and additional details of epidemiology, seroprevalence, HCV RNA positivity figures, treatment availability etc.

## Results

### Search results

A total of 99 abstracts for manuscripts published between September 1990 and June 2016 were identified. Of these, 42 contained information relevant for our review and full texts were retrieved [11, 14–60] (Table 1 and Fig. 1). The majority of publications (73.8%, 31/42) assessed the prevalence of HCV infection specifically in high-risk groups, including HIV infected patients, injecting drug users (IDU), community sex workers (CSW), men that have sex with men (MSM) and renal haemodialysis patients [11, 17, 21–23, 25, 26, 28, 29, 31, 32, 34, 36, 37, 39, 41–47, 51–53, 55–60] (Table 1). Online search for Vietnamese articles retrieved one research publication and four Standard Operating Procedures (SOPs); only the Vietnamese research paper retrieved from a Vietnamese journal was included in this analysis [38].

**Table 1:** Characteristics population-based studies reporting prevalence of HCV in Vietnam

Year	First Author	Prevalence	Group Type	Sample Size	Reference
1998	Kakumu	1%	General Population	1179	[47]
2007	Nguyen	1%	General Population	837	[45]
2015	Quesada	4.7%	General Population	27/571	[27]
2015	Do	3.3% 1.3%^*^	General population	509	[30]
2003	Tran	2%	General population	334	[15]
1996	Corwin	2%	General population	188	[49]
2012	Sereno	0.38–4.3%	General population	NA^b^	[21]
2016	Martinello	42.5%	HIV	89.452^a^	[24]
2015	Nadol	53.3%	HIV/IDU	3010	[32]
2015	Zang	88%	HIV	1434	[29]
1999	Follézou	>80%	HIV	280	[18]
2012	Durier	22.9%	HIV	110	[20]
2016	Nguyen	89%	HIV	104	[26]
2016	Hser	74%	HIV/IDU	NA^b, c^	[11]
2012	Sereno	95.8%	HIV/IDU	NA^b^	[21]
2012	Gish	87%	IDU	NA^b^	[36]
1998	Kakumu	47%	Liver Disease	1179	[47]
2003	Tran	10%	Liver disease	334	[15]
1996	Corwin	10%	Liver Disease	188	[49]
2004	Buchy	9%	Liver disease	45	[46]
2012	Gish	23%	Liver disease	NA^b^	[36]
1993	Cordier	2%	Hepatocarcinoma	152	[54]
2010	Bjoerkvoll	12.7%	Blood donors	1305	[38]
2012	Viet	76.4%	Blood Donors	1200^a^	[40]
1994	Song	20.6%	Blood Donors	491	[52]
2012	Dunford	26.6%	Dialysis	8652^a^	[37]
2016	Duong	8%	Dialysis	142	[22]
2015	Duong	6%	Dialysis	113	[31]
2012	Gish	54%	Hemodialysis	NA^b^	[36]
2016	Nadol	28.4%	MSM	1588	[25]
2012	Dunford	8.7%	CSW	8652^a^	[37]

^*^Presence of HCV RNA

^a^Multi countries study

^b^ *NA* Not applicable

^c^Estimate

Twenty one percent (9/42) of the publications reported data from Ho Chi Minh City (HCMC) or from the southern Vietnam region (Table 1), while 52.3% (22/42) reported studies conducted in the north of the country, principally in Hanoi and surrounding provinces [18, 22, 27, 32, 36, 47, 49, 52, 53] (Table 1). Eight of forty two retrieved publications characterized the diversity of HCV genotypes in Vietnam [14, 16, 21, 33, 34, 48, 50, 51] (Table 1). Two manuscripts were reviews assessing risk factors associated with HCV acquisition in Vietnam [11, 24]. One article, retrieved from a Vietnamese website assessed the prevalence of HCV in the Vietnamese ethnic minority [38].

Nine clinical trials were found registered with *ClinicalTrials.com* (see Table 2). Five out of nine were completed but no results have been published to-date, two are completed with results available, one is active but no recruiting, while one is actively recruiting (Fig. 1). Only the two clinical trials that had published results were included in the review, the remaining seven studies have been excluded.

**Table 2:** List of the clinical trials conducted in Vietnam to date ^a^

Year	Title	City	Sponsor	Status
2010–2016	Hepatic Safety of Raltegravir Versus Efavirenz as HIV Therapy for Patients With HIV and HCV Coinfection	Ho Chi Minh City, Hai Phong	University of Hawaii	Active, not recruiting
2014–2016	The Study of Safety, Pharmacokinetics, Pharmacodynamics of Peglamda (Peginterferon Lamda 1) on Healthy Volunteers and the Preliminary Evaluation of Peglamda and Hepasig (Ribavirin) Treatment’s Effects on Chronic Hepatitis C Patients	Unknown	Nanogen Pharmaceutical Biotechnology Co., Ltd	Completed
2015–2016	Feasibility of Interventions on People Who Inject Drugs in Vietnam			
	Implementation of a Sexual Health Intervention for Young Men Who Have Sex With Men (MSM) in Two Vietnamese Cities	Ho Chi Minh City, Hanoi	Inserm-ANRS	Completed
2013–2016	HCV Treatment in HIV Co-Infected Patients in Asia	Hanoi	amfAR,	Completed
2016	Long Term Follow-up Study to Assess Durability of Sustained Virologic Response in Alisporivir-treated Hepatitis C Patients	Unknown	Debiopharm International SA	Completed, has results
2011–2016	Efficacy and Safety of Alisporivir Triple Therapy in Chronic Hepatitis C Genotype 1 Treatment-naïve Participants	Unknown	Debiopharm International SA	Completed, has results
2016–2017	Efficacy and Safety of Sofosbuvir/Velpatasvir Fixed Dose Combination for 12 Weeks in Participants With Chronic HCV	Unknown	Gilead Sciences	Recruiting
2015	3-year Follow-up Study to Assess the Viral Activity in Hepatitis C Patients Who Failed Feeder DEB025/Alisporivir Study		Novartis Pharmaceuticals	Completed
2014–2016	Implementation of a Sexual Health Intervention for Young Men Who Have Sex With Men (MSM) in Two Vietnamese Cities	Hanoi	National Development and Research Institutes, Inc.	Completed

^a^For further details please see the following website: ClinicalTrials.gov

### The epidemiology of hepatitis C in Vietnam

**Fig. 2:**
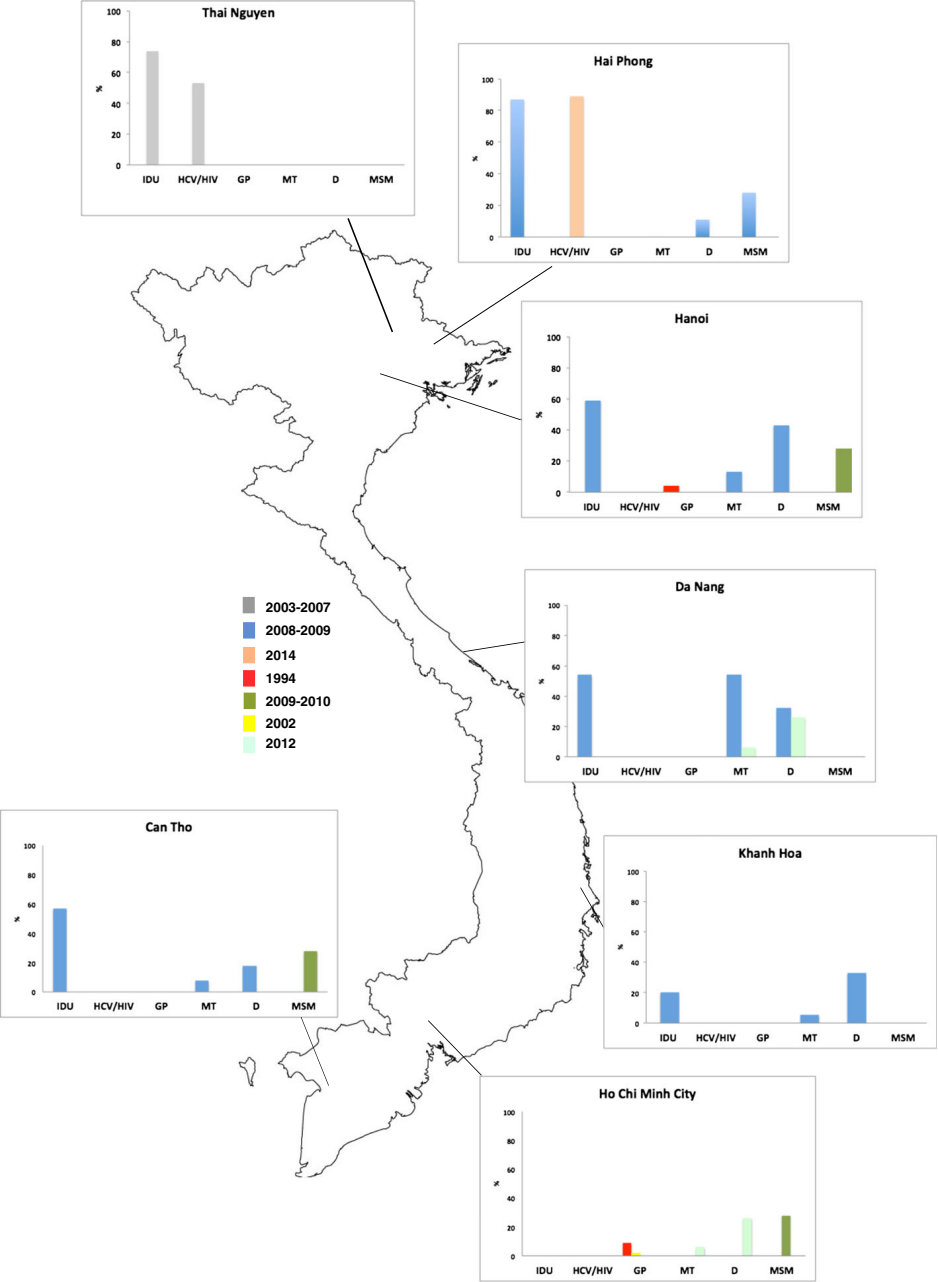
Vietnam Map and summary of the main studies conducted so far A summary of the main studies performed in Vietnam to-date measuring the prevalence of HCV viral detection (PCR positivity) in selected populations by year of study publication and geographic location. * Data for ‘General adult healthy populations’ is for measured anti-HCV antibody seroprevalence. The map represents the location where the main studies have been conducted and summarized the HCV prevalence or seroprevalence observed during the studies in different groups, such as IDU, MSM, liver disease, hemodialysis and multi-blood transfusion and general population

In Vietnam, the background prevalence of HCV infection in the general population, at least historically, appears to vary depending on the region studied. In 1994 it was estimated that the seroprevalence in individuals without liver disease was 9% (43/491; 95% CI, 6.4%–11.5%) and 4% (18/511; 95% CI, 2.3%–5.6%) in HCMC and Hanoi, respectively [53] (Fig. 2). By 1998 it was estimated that in HCMC, the HCV seroprevalence in patients with underlying liver disease was 23% (69/289; 95% CI, 18.1%–27.8%) and in this population, HCV RNA was detectable in blood of 61% (42/69; 95% CI, 49.4%–72.5%) of the individuals [47]. This same study reported an HCV seroprevalence in healthy individuals in Da Lat (in the Southern Highlands) of 1% (9/890; 95% CI, 0.9%–1.0%); HCV RNA was detected in 44% (4/9) of these individuals [47]. Four years later in 2002, HCV RNA detection in HCMC was estimated to be 2% (2/100; 95% CI, 1.3%–5.3%) in apparently healthy individuals and 33% (45/234; 95% CI, 27.0%–39.0%) in patients with liver disease [15]. In Binh Thuan province (212 km northeast of HCMC) HCV seroprevalence in the general population between 2005 and 2007 was estimated to be 3.3% (17/509; 95% CI, 1.7%–4.8%); the male population exhibited a higher burden of seropositive samples (3.3%) than women (1.8%), and HCV RNA was detected in 52.9% (9/17) of the identified anti-HCV positive individuals (Fig. 2) [30]. Furthermore, one article was retrieved from a Vietnamese journal. The original manuscript was in Vietnamese and only the abstract was translated in English. The manuscript assessed the seroprevalence of HCV in the Vietnamese ethnic minority in the north of Vietnam and the detected seroprevalence in 1305 individuals was 1.22% (95% CI, 0.6%–1.7%) in 2011 [38].

The majority of the remaining publications evaluated HCV prevalence in specific high-risk groups (Table 1). These data estimated that 89% (1255/1434; 95% CI, 87.3%–90.6%) of IDU in Nguyen province (Northern Vietnam) were HCV RNA positive between 2005 and 2007 and 35% (502/1434; 95% CI, 32.5%-37.4) were HIV/HCV co-infected [29]. Similar figures were reported in Hai Phong province (northern Vietnam) in 2014 (Fig. 2), where the prevalence of HCV in HIV-infected individual was 89.4% (93/104; 95% CI, 82.1%–96.6%). This study also reported that the majority of HCV cases presented with clinical features of advanced liver fibrosis and cirrhosis [26].

Between 2005 and 2012, several studies were conducted around Vietnam (including HCMC, Hanoi, Hai Phong Province, Da Nang, Khanh Hoa Province and Can Tho), which aimed to determine HCV seroprevalence in patients undergoing renal haemodialysis or receiving multiple blood transfusions [23, 37]. These six hospital-based studies found an apparent iatrogenic HCV seroprevalence of 26% (*n* = 153/575; 95% CI, 22.4%–29.5%) and was similar at all sites assessed.

In 2010, a study estimated the prevalence of HCV infection in MSM in the south of Vietnam (Can Tho and HCMC) and in the north of Vietnam (Hai Phong Province and Hanoi) [25]. The overall seroprevalence of infection in 1588 male participants was 28.4% (95% CI, 26.1%–30.6%) [25]; 29% of the individuals were co-infected with HIV and more northerly regions observed a higher HCV and HCV/HIV co-infection prevalence [25]. A single publication from 1997 assessed the frequency of HCV infection in patients with chronic hepatitis in HCMC, and concluded that HCV was the underlying etiology in 19–27% of chronic hepatitis cases [19]. However, no indication of the sample size used to derive this estimate was provided.

### The molecular epidemiology of HCV in Vietnam

A study conducted in four major conurbations in Vietnam – Hanoi, Hai Phong Province, Da Nang, Khanh Hoa Province and Can Tho, identified HCV genotypes 1, 6, 3 and 2 in 59.9% (*n* = 169), 37.9% (*n* = 107), 1.8% (*n* = 5) and 0.4% (*n* = 1) of samples collected from mixed healthy and high-risk populations, respectively [37]. While genotype 1 was the most prevalent HCV genotype in all four areas, the genotype distribution ranged from 47% in Khanh Hoa Province to 81% in Da Nang [37]. These four genotypes (1–3, 6, and) were identified in dialysis and multi-transfused individuals with nine recognized subtypes (1a, 1b, 2a, 3a, 3b, 6a, 6e, 6 h, 6 l) [39]. A further study similarly identified HCV genotype 1 as the predominant virus circulating in MSM in Hanoi, Hai Phong Province, HCMC and Can Tho [25]. Additionally, plasma samples from 97 HCV infected individuals were molecularly characterized between 2003 and 2010 in HCMC. The RNA sequences obtained from these samples suggested that genotypes 1b and 2a were imported from East Asia, 1a from the United States, 2i, 2j and 2 m imported from France [33, 58]. They also identified an additional 4 subtypes of genotype 6 (6a, 6ax, 6xb and 6xc) with no obvious international link [34].

### Clinical trials

Of the nine trials registered on *ClinicalTrials.gov*, only two had published results available at time of review [61, 62]. Both studies were sponsored by Debiopharm International SA and neither the source country information nor the locations where trial participant recruitment was performed within the country have been specified. Both studies were multicenter international trials to assess the treatment responses (SVR to single agent or combination treatment) to the synthetic cyclosporine-like agent, Alisporivir, a novel host-targeting antiviral (HTA) agent [61, 62].

## Discussion

Our literature search identified a paucity of published data regarding the nature or burden of HCV infection in Vietnam. Studies in the publications identified were generally conducted in high-risk populations and focused in the more populous northern provinces (around Hanoi) and southern provinces (around HCMC) of the country. In addition, most studies have measured HCV antibody seroprevalence rather than HCV RNA prevalence, which would more reliably indicate the numbers of individuals with active infection. As elsewhere, the available literature suggested that blood transfusions, intravenous drug use, and unsafe therapeutic injections were a likely common source of HCV infection in the Vietnamese populations studied. Furthermore, the data suggests while the HCV seroprevalence in the general population is approximately 4% (18/511; 95% CI, 2.3%–5.6%) in Hanoi and 9% (43/491; 95% CI, 6.4%–11.5%) in Ho Chi Minh City, which is high in comparison to America or Europe [63, 64], this is likely to be even higher in IDU (55.6%, *n* = 556/1000; 95% CI, 52.8%–58.3%), MSM (38.8%; *n* = 1558; 95% CI, 36.3%–41.2%), and hemodialysis patients (26.6%, *n* = 153/575; 95% CI, 22.4%–29.5%) [23, 31, 53, 57] . There are still substantial gaps in our understanding of the molecular epidemiology of HCV infection in Vietnam, however the limited findings from the few studies that have been conducted suggest that multiple (≥4) HCV genotypes and subtypes are circulating in the population [25, 37].

## Ascertaining the burden of hepatitis C in Vietnam

Ascertaining the true likely burden of infection in Vietnam would facilitate advocacy for the implementation of disease specific strategies and guidelines, and provide useful baseline data for further epidemiological measurements. Our search identified that HCV seroprevalence in the population may be high (1–4%) compared to other countries in the region [3, 27, 53]. For example, several large-scale seroprevalence studies have reported HCV estimates of asymptomatic Chinese ranging from 0.39 to 3.2% depending of the region [65]. Other Asian countries such as Japan, India or Indonesia reported an HCV seroprevalence of between 2.3, 0.9 and 2.1% respectively [4].

Observations supporting the speculation that the true seroprevalence might be underestimated include data from neighboring countries such as China, where the reported rate of HCV infections increased more than 10-fold (from 21,000 reported cases/year to 210,000 reported cases/year) between 2003 and 2011 [66]. Reasons for this increase may include an increase in life expectancy and/or the increased detection/occurrence of new infections due to better surveillance and improved diagnostics. The ageing of the population creates two potentially major pressures on health care finances: increased utilization of health services and decreased revenues (as a declining share of the population is economically active).

Further insights into the current HCV situation in Vietnam may be provided by two non-resident populations; data on HCV infection in USA veterans after the Vietnam/American war suggest that returning veterans had a higher risk (10–17%) of being HCV infected than the general North American population (1.3%) [63, 64, 67]. This conflict was associated with the highest rate of HCV acquisition in comparison to any that the US has been engaged in. More recently, it was observed that Vietnamese migrants to other countries have a higher prevalence (5.8%) of HCV infection when compared to the autochthonous population (1.3%) [60, 68].

The transmission of HCV infection in clinical settings is still particularly concerning in Vietnamese healthcare facilities; the high HCV prevalence (26%) in haemodialysis patients [23, 31, 57] has not been observed in other Asian-Pacific countries (0.7–18.1%) [69]. This literature review also found a high seroprevalence (27%) in other high-risk populations including IDU, MSM, and CSW, similar data has been reported from studies conducted between the Myanmar and Chinese border region [70].

We therefore speculate that the seroprevalence in the general population might be higher than that currently estimated as the limited data currently available (only 42 publications published in over two decades) is from studies performed in only a few locations across Vietnam and which mostly had relatively small sample sizes upon which to base these calculations. Of note, the two most recent and larger studies performed to estimate seroprevalence in the general population have found an HCV prevalence of 3.3 and 4.7%, respectively [27, 30].

### Identifying the genetic characteristics of HCV to improve public health

In addition to assessing the burden of HCV disease, further data is required regarding HCV genotype, viral mutations and viral loads in HCV infected patients in order to assess how to implement the recent HCV treatment guidance, and to estimate the cost-benefit of an integrated HCV control program.

While currently available data are limited, they imply a broad genetic diversity within circulating HCV strains in all parts of the country [33, 34, 37, 39, 58]. More detailed phylogenetic approaches have proposed that the HCV population has been influenced by the international turmoil of the past, with importation of genotypes that are more typically associated with North America, Europe and other parts of Asia [34]. This genetic variation can also be observed in high-risk groups, meaning that the formation of a local treatment policy may be more complex than in other locations. For example, the distribution of HCV genotype in haemodialysis patients is variable according to the geographical region, this may be due to a lack of infection control and or point source outbreaks [37]. This genotype distribution is in contrast to other countries in Asia, such as China, where hemodialysis patients are predominantly affected by genotype 2 HCV [71, 72]. An improved understanding of the genotype distribution and frequency of viral treatment mutations among Vietnamese patients will facilitate the development of appropriate local guidelines.

### Current treatment options, molecular challenges and social taboos

Early diagnosis and access to treatment are essential components for an effective HCV control program in Vietnam. As in most LMICs, current treatment for HCV infection in Vietnam is limited to interferon-based (IFN-gamma and pegylated-IFN) therapy [10]. More recently, some patients have been treated with newer DAA agents at the Hospital for Tropical Diseases (HTD) in HCMC, where more than 18,000 patients with viral hepatitis are assessed each year. Further clinical treatment studies have received funding and are planned to commence in the near future [73].

The more widespread introduction of the DAAs is hindered by a lack of data regarding HCV genotype distribution and viral treatment mutations [74]. This lack of data is especially problematic for the early detection of treatment resistance mutations, which occurred previously with hepatitis B treatment and HCV treatment in Japan and North America [75–77]. Furthermore, the efficacy of DAAs against some of the less common genotypes, such as genotype 6 which was frequently identified in previously studies, is not well established [78].

Other barriers to increasing the utilization of HCV treatment in resource-limited settings such as Vietnam include high costs, a perceived complexity in treatment regimes and bureaucratic support for importation, licensing and distribution of medicines for which specific clinical trial data in the local populations is lacking. Vietnam is a rapidly developing country, whose GDP per-capita has increased from $1095 to $1684 from 2004 until 2014 [79]. However, the per capita income remains low (~ $1024) [80] with limited annual spending on healthcare (according to WHO, approximately $264 in 2006) [80]. A large proportion of people in Vietnam do not invest in health insurance and even those who do may not have full coverage to expensive treatment courses: pegylated-interferon courses are reimbursed at 30% ($10,000–18,000 for a 48 week treatmentcourse), and this partial reimbursement is based on doctor opinion only, with no allowance given for those in high-risk populations such as haemodialysis patients [81, 82].

While the treatment of HCV infection requires prescription from a medical practitioner, a further concern is the non-completion of treatment courses due to high costs and changes in family/patient financial circumstances. Some patients may also travel to other countries in the region where medications, which may be substandard, might be more affordable. Incomplete or inadequate treatment courses are likely to lead to increased rates of viral resistance, which has been previously widely observed in hepatitis B treatment programs in Vietnam [83, 84]. Further, PEG + INF + RBV remains (as 2016) the first line of therapy for chronic patients in many part of Asia, Vietnam included [85].

### Study limitations

The majority of the studies included here had small sample sizes and concentrated on groups of individuals at known high-risk of acquiring HCV infection thus risking an overestimation bias. Given the heterogeneity of the study populations sampled, in addition to the broad time period and diverse geographic locations described, more accurate meta-analysis is not currently feasible. Thus, a considerable knowledge gap remains regarding the likely true burden of chronic HCV infection in the general, healthy adult Vietnamese population.

## Conclusions

Relatively little is known regarding the burden of HCV infection in the general population in Vietnam. Available data suggest that the seroprevalence could be higher than previously assumed, especially in known high-risk groups such as patients undergoing haemodialysis. Limited data predict a broad distribution of HCV genotypes and, to-date, very few HCV treatment trials have been performed in Vietnam. While HCV control programs in Vietnam are in their infancy, there is an urgent need for more detailed disease burden and viral genotyping data, which, in addition to planned treatment studies, could be used to inform the development of local HCV treatment strategies and thus eventual attainment of the 2030 elimination target.

## Abbreviations

HCV: Hepatitis C virus
DAA: Direct acting anti-viral
HTD: Hospital for Tropical Diseases
HCMC: Ho Chi Minh City
IFN: Interferon-based
LMIC: Low to middle income countries
WHO: World Health organization
IDU: Injecting drug users
MSM: Men that have sex with men
CSW: Commercial sex workers
SVR: Sustained virological response
